# Dietary Microbial Polysaccharides in Food Systems: Production, Functional Properties, and Applications

**DOI:** 10.1002/fsn3.72395

**Published:** 2026-09-21

**Authors:** Hye‐Bin Lee, Yu‐Rim Chae, Yu Ra Lee, Yoonsook Kim, Ho‐Young Park

**Affiliations:** ^1^ Food Functionality Research Division Korean Food Research Institute Wanju‐gun Republic of Korea; ^2^ Department of Food Biotechnology University of Science and Technology Daejeon Republic of Korea

**Keywords:** dietary fiber, exopolysaccharides, food applications, lactic acid bacteria, structure–function relationships

## Abstract

Microbial polysaccharides are structurally diverse biopolymers produced by bacteria, fungi, yeasts, and algae, functioning as extracellular matrices and/or intracellular carbon reserves depending on the organism and growth conditions. Among them, microbial exopolysaccharides (EPSs) have attracted substantial interest because their tunable architectures confer desirable techno‐functional attributes alongside emerging biofunctional potential. This review synthesizes recent advances in EPS research with emphasis on how microbial origin, biosynthetic routes, and glycosidic linkage patterns shape higher‐order structure and, ultimately, functional performance in food systems. We summarize major EPS classes and representative producing microorganisms, outline key structure–property relationships relevant to formulation and processing, and critically discuss reported biological activities in the context of evidentiary strength and translational relevance. In addition, we compare current production, recovery, and purification strategies, highlighting practical considerations for scalability, product consistency, and regulatory compliance. Finally, we identify opportunities and bottlenecks for next‐generation dietary EPSs, including precision fermentation, strain engineering, and application‐driven design to meet clean‐label and sustainability goals. Collectively, this review provides a framework for rational selection and development of microbial EPSs as functional ingredients for food innovation.

## Graphical abstract

Microbial polysaccharides from fungi, bacteria, and microalgae exhibit diverse structures and biological activities. Their antioxidant, antimicrobial, immunomodulatory, and prebiotic properties highlight their potential as functional ingredients for food applications.

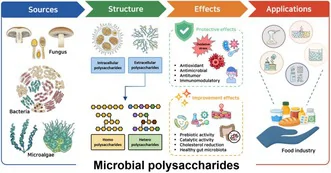

## 1 Introduction

Dietary fiber comprises non‐digestible carbohydrate polymers and associated substances in plant‐based foods that resist digestion and absorption in the upper gastrointestinal tract and are subsequently fermented by the colonic microbiota (Fu et al. 2022). Through microbial fermentation, dietary fibers can reshape community metabolism and generate bioactive fermentation products, including short‐chain fatty acids (SCFAs) and other metabolites (Koh et al. 2016). These metabolites are increasingly linked to host‐relevant outcomes such as improved barrier function and protection against intestinal inflammation (Moncada et al. 2024). Importantly, when a dietary substrate is selectively utilized by host microorganisms and confers a health benefit, it is classified as a prebiotic (Gibson et al. 2017; Rezende et al. 2021).

Lactic acid bacteria (LAB), which produce lactic acid as a major metabolic end product, are widely distributed in nature, including plant materials, milk, and meat, and are generally recognized as safe strains (Liu et al. 2021). Beyond LAB, a range of industrially relevant microorganisms, including *Xanthomonas* and *Sphingomonas*, produce structurally diverse microbial polysaccharides with established techno‐functional value in food systems (Abdl Aali and Al‐Sahlany 2024; Nsengiyumva and Alexandridis 2022). In this review, we use the term exopolysaccharides (EPSs) to refer to extracellularly secreted, high‐molecular‐weight polysaccharides produced by microorganisms, including LAB‐derived EPSs, some of which have been reported to exhibit prebiotic‐like or gut‐relevant bioactivities (Tarique et al. 2023). This definition should be distinguished from extracellular polymeric substances, which describes the broader composite biofilm matrix (polysaccharides, proteins, nucleic acids, and lipids) rather than the polysaccharide fraction alone.

From a microbial perspective, EPS biosynthesis can confer ecological advantages by enhancing resilience to environmental stressors and contributing to cell–cell and cell–surface interactions. EPSs can protect cells under adverse conditions and facilitate adherence and biofilm formation, thereby supporting persistence in fluctuating environments (Dertli et al. 2015; Nwodo et al. 2012; Singh et al. 2021). These same attributes underpin the functional relevance of EPSs in food matrices, where polymer architecture, including monomer composition, branching, and glycosidic linkages, can be translated into formulation‐relevant properties such as viscosity, gelation, water binding, stabilization, and texture.

Microbial polysaccharides are high‐molecular‐weight carbohydrates synthesized by various microorganisms, including bacteria, fungi, yeasts, and algae (Qamar et al. 2022). These polysaccharides include both intracellular storage polymers, such as glycogen that serves as an energy and carbon reserve, and extracellularly secreted polymers with diverse functional roles (Khan et al. 2022). Owing to their versatile physicochemical and biological properties, microbial extracellular polysaccharides are widely utilized in the food industry and have also found applications in cosmetic and pharmaceutical sectors (Korcz et al. 2018). In particular, EPSs such as xanthan gum, gellan gum, dextran, and LAB‐derived EPSs have attracted focused research interest due to their prospective applications as texturizing and functional ingredients in food systems (Korcz and Varga 2021; Prete et al. 2021; Rajoka et al. 2020).

Several comprehensive reviews have summarized microbial exopolysaccharides with emphasis on specific microbial groups, individual polysaccharides, or their biological functions (Prete et al. 2021; Korcz and Varga 2021). Building on these previous studies, this review presents an integrated perspective on dietary microbial polysaccharides by linking microbial origin, structural characteristics, production strategies, and food applications within the context of food systems. Particular emphasis is placed on the relationships between structural characteristics and the techno‐functional properties of microbial polysaccharides relevant to food applications.

## 2 Microorganisms for Dietary Polysaccharide Production

EPS‐producing bacteria are found across a diverse range of phylogenetic groups, including both Gram‐negative and Gram‐positive groups (Dogsa et al. 2024; Sutherland 2001b). Representative EPS‐producing taxa include Gram‐negative classes such as *Alphaproteobacteria*, *Betaproteobacteria*, and *Gammaproteobacteria*, and Gram‐positive classes such as *Bacilli*, *Clostridia*, and *Actinomycetia*. Representative EPS‐producing bacterial groups and genera are summarized in Table 1.

**TABLE 1:** Representative EPS‐producing bacteria across major phylogenetic groups.

Gram status	Taxonomic class	Representative genera	References
Gram‐negative	Alphaproteobacteria	*Acetobacter*	Upadhyaya et al. (2025)
		*Gluconobacter*	
		*Gluconacetobacter*	
		*Komagataeibacter*	
		*Kozaki*a	
		*Neoasaia*	
		*Agrobacterium*	
		*Rhizobium*	
		*Zymomonas*	
	Betaproteobacteria	*Alcaligenes*	Barcelos et al. (2019)
		*Achromobacter*	
	Gammaproteobacteria	*Azotobacter*	Barcelos et al. (2019)
		*Pseudomonas*	
		*Enterobacter*	
		*Alteromonas*	
		*Pseudoalteromonas*	
		*Xanthomonas*	
		*Halomonas*	
		*Erwinia*	
		*Vibrio*	
		*Klebsiella*	
Gram‐positive	Bacilli	*Bacillus*	Aziz et al. (2024)
		*Paenibacillus*	
		*Lactobacillus*	
		*Leuconostoc*	
		*Streptococcus*	
	Clostridia	*Sarcina*	Netrusov et al. (2023)
	Actinomycetia	*Bifidobacterium*	Netrusov et al. (2023)
		*Rhodococcus*	

## 3 Classification of Microbial Polysaccharides by Cellular Location

### 3.1 Intracellular Polysaccharides

Although this review primarily focuses on extracellular polysaccharides because of their established relevance to food systems, intracellular polysaccharides (IPSs) are briefly discussed to provide a broader overview of microbial polysaccharide diversity. IPSs are predominantly sourced from fungi, including species such as *Eurotium cristatum*, *Pleurotus geesteranus*, and *Fomitopsis pinicola* (Hao et al. 2016; Song et al. 2021; Zhang et al. 2024). As shown in Table 2, several IPSs have been reported to exhibit biological activities, including antioxidant, antitumor, and immunomodulatory effects (Khan et al. 2022); however, these findings should be interpreted cautiously in the context of food applications.

**TABLE 2:** Effects and applications of microbial intracellular polysaccharides.

Organism	Effects	Application	References
*Eurotium cristatum*, *Pleurotus geesteranus*, *Fomitopsis pinicol*a	Antioxidant, antitumor, immunomodulatory	Biomedical, pharmacological	Khan et al. (2022)
*Armillaria luteo‐virens*	Immune protection, cytokine secretion	Immunotherapy, immune modulation	Li et al. (2023)
*Pleurotus eryngii*	Hepatoprotective, antioxidant, reducing serum triglyceride & albumin, increasing bilirubin	Industrial biotechnology, biomedical	Zhang et al. (2016)

For example, IPSs isolated from the fruiting bodies of *Armillaria luteo‐virens* have been reported to protect immune organs and increase serum cytokine levels, consistent with immunomodulatory activity (Li et al. 2023; Liu et al. 2024). In *Pleurotus eryngii*, Zhang et al. identified two IPS fractions (IPS‐1 and IPS‐2) and evaluated their hepatoprotective and antioxidant‐related effects (Zhang et al. 2016). IPS‐2 showed stronger hepatoprotective activity, accompanied by reductions in serum triglyceride and albumin levels, while serum bilirubin increased; together, these changes were interpreted as reflecting improved hepatic antioxidant status. Collectively, these studies demonstrate that fungal IPSs possess diverse biological activities in preclinical models, supporting their potential as bioactive microbial polysaccharides. However, current evidence remains largely limited to in vitro and animal studies, and further investigation is required to clarify their relevance and applicability in food and nutrition contexts.

### 3.2 Extracellular Polysaccharides

**FIGURE 1:**
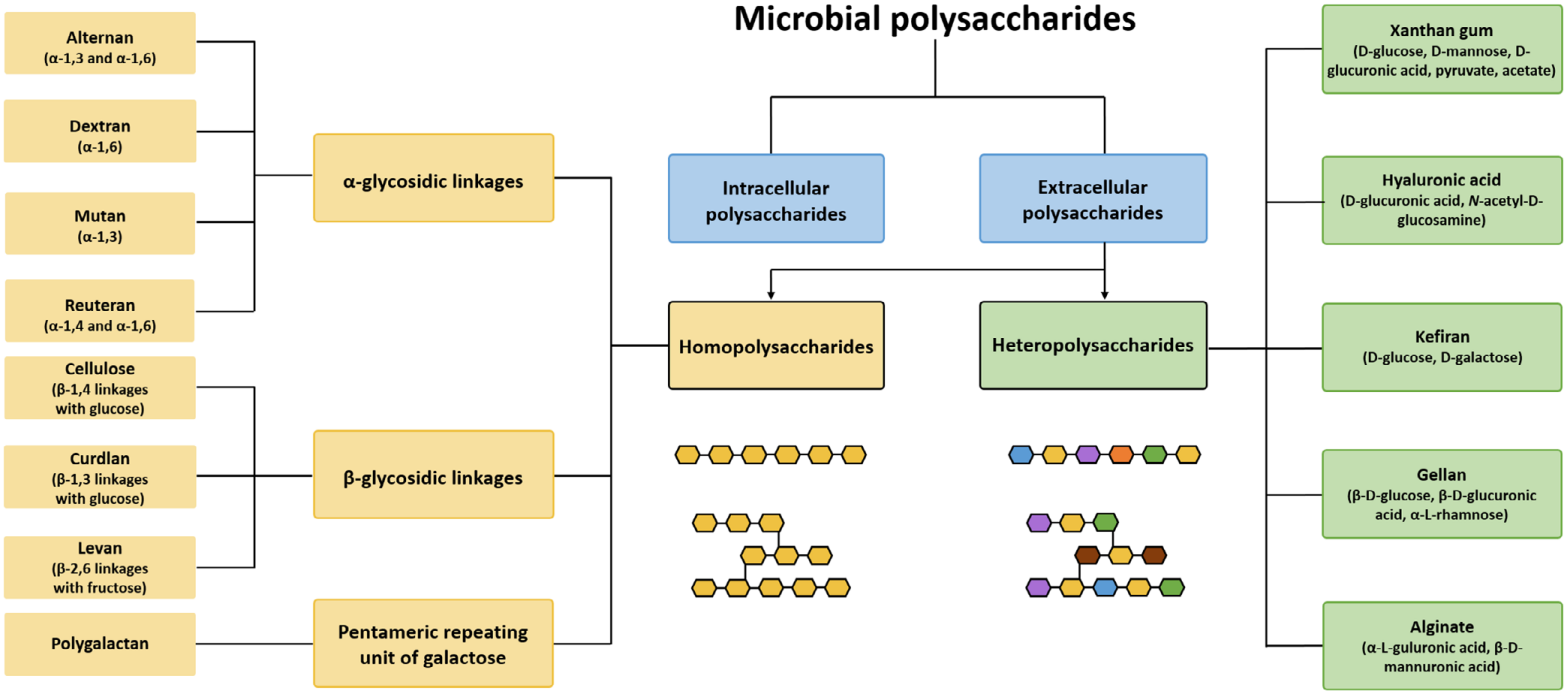
Classification of microbial polysaccharides. Microbial exopolysaccharides can be categorized according to their spatial distribution, either intracellularly or extracellularly. Extracellular polysaccharides may consist of repeating units of identical monosaccharides, referred to as homopolysaccharides, or they may be composed of two or more distinct monosaccharide units, known as heteropolysaccharides.

The term “exopolysaccharide” denotes a polysaccharide secreted outside the microbial cell: “exo,” which denotes “external,” and “polysaccharide,” which refers to a carbohydrate composed of multiple sugar monomers (Kaur and Dey 2023; Singh et al. 2019). In microorganisms, EPSs contribute to extracellular matrices, cell protection, and interactions with surrounding environments (Osemwegie et al. 2020). EPSs are typically high‐molecular‐weight biopolymers, with molecular weights ranging from 10 to 1000 kDa (Schmid et al. 2015). Microbial EPSs may support intestinal health by modulating microbial communities, immune responses, and barrier‐related functions (Chaisuwan et al. 2020). Based on their chemical composition and biosynthetic pathways, EPSs can be categorized into two primary groups: homopolysaccharides (HoPS) and heteropolysaccharides (HePS; Figure 1) (Saadat et al. 2019). HoPS, including cellulose, dextran, and pullulan, comprise a single type of monosaccharide residue (Khan et al. 2022). In contrast, HePS, such as xanthan gum and hyaluronic acid, contain two or more types of monosaccharide residues (Yildiz and Karatas 2018). Bacteria synthesize EPSs as a defensive mechanism against phagocytosis and environmental stressors (Nwodo et al. 2012). Among them, EPSs synthesized by LAB have attracted particular attention due to their beneficial effects on host health and their potential applications as food ingredients (Zhang, Xiao, et al. 2023).

#### 3.2.1 HoPS

HoPS produced by LAB are typically composed of repeating units of either D‐glucose or D‐fructose and often reach high molecular weights (approximately 10^5^–10^7^ Da) (Caggianiello et al. 2016; Pérez‐Ramos et al. 2016). Compared with HePS, HoPS are generally produced at higher yields, largely because many are synthesized extracellularly by sucrase‐type enzymes using sucrose as the donor substrate. HoPS diversity arises from differences in backbone architecture, branching frequency, and linkage specificities, which in turn govern solubility and functional performance in food matrices (Ruas‐Madiedo and De Los Reyes‐Gavilán 2005).

Alternan is a branched glucan with high water solubility and consists primarily of glucose residues linked by α‐(1 → 6) and α‐(1 → 3) glycosidic bonds (Table 3). Alternan is synthesized by alternansucrase using sucrose as the substrate (Wangpaiboon et al. 2018), and has been reported to be produced by *Leuconostoc mesenteroides* (Leathers et al. 2004; Zannini et al. 2016). In food‐related applications, alternan has been described as a prebiotic candidate and as a bulking/food‐extending ingredient (Zannini et al. 2016).

**TABLE 3:** Effects and applications of microbial extracellular polysaccharides.

Organism	Polysaccharide	Major monosaccharides	Linkage	Effects	Food‐related applications	References
*Leuconostoc mesenteroides*	Alternan	Glucose	α‐(1 → 3), α‐(1 → 6)	Prebiotic	Bulking agent, food extenders	Zannini et al. (2016)
*Weissella*, *Liquorilactobacillus*, *Leuconostoc mesenteroides*	Dextran	Glucose	α‐(1 → 6)	Antioxidant, cholesterol reduction, immunomodulatory, antitumor	Improve the rheology and texture of many fermented food products	De Bellis et al. (2022)
*Streptococcus mutans*	Mutan	Glucose	α‐(1 → 3) with α‐(1 → 6) side chains	Dental biofilm formation and stability	Oral health management	Angelin and Kavitha (2020); Bowen and Koo (2011)
*Limosilactobacillus reuteri*	Reuteran	Glucose	α‐(1 → 4), α‐(1 → 6)	Extended shelf life, prebiotic activity	Use in bakery to enhance softness	Korcz and Varga (2021)
*Komagataeibacter* spp.	Cellulose	Glucose	β‐(1 → 4)	Antimicrobial, antioxidant, catalytic activity	Thickener, a suspending or stabilizing agent in foods, ingredient in fiber‐enriched low‐calorie & low‐cholesterol diets, food packaging	Netrusov et al. (2023)
*Alcaligenes* spp., *Agrobacterium* spp., *Paenibacillus* spp.	Curdlan	Glucose	β‐(1 → 3)	Healthy gut microbiota, prevent obesity	Food additives in the dairy industry, reduce calories	Chaudhari et al. (2021)
*Bacillus* spp., *Halomonas* spp., *Pseudomonas* spp., *Aerobacter* spp., *Streptococcus* spp., *Microbacterium* spp.	Levan	Fructose	β‐(2 → 6) with β‐(2 → 1) branching	Hair care, lower cholesterol, prebiotics, anti‐irritant, antioxidant, anti‐inflammatory	Aquaculture, pharmaceuticals, inhibition of metal corrosion, renewable transportation fuel	Öner et al. (2016)
*Lactococcus lactis* subsp. *lactis*, *Lactobacillus delbrueckii* subsp. *bulgaricus*	Polygalactan	Galactose	Pentameric repeating unit of galactose	Limited specific evidence available	Limited food applications reported	Jurášková et al. (2022)
*Xanthomonas campestris* , *Xanthomonas phaseoli* , *Xanthomonas malvacearum*	Xanthan	D‐Glucose, D‐mannose, D‐glucuronic acid, pyruvate, acetate	Celluloselike backbone of β‐(1 → 4)‐linked glucose units	Antimicrobial, antioxidant, electromagnetic properties, catalytic activity	Thickener, stabilizer, suspending agent, food packaging	Revin et al. (2023)
*Pasteurella multocida* , *Streptococcus* spp.	Hyaluronic acid	D‐glucuronic acid, *N*‐acetylglucosamine	β‐(1 → 4)‐D‐Glucuronic acid and β‐(1 → 3)‐*N*‐acetyl‐D‐glucosamine disaccharide	Activate skin cells, improve the essential nutrients for the eyes, skin, collagen, immunity	Taste enhancer, versatile packaging material	Cheng et al. (2023)
*Sphingomonas elodea*, *Sphingomonas pseudosanguinis*	Gellan	L‐Rhamnose, D‐glucose, β‐D‐glucuronate	Repeating tetrasaccharide units consisting of β‐(1 → 3)‐D‐glucose, β‐(1 → 4)‐D‐glucuronic acid, α‐(1 → 4)‐L‐rhamnose	Antioxidant, antitumor properties	Food packaging, coatings for fresh produce, flavor films, improvement of texture, edible strips and wrappers	Abdl Aali and Al‐Sahlany (2024)
*Lactobacillus kefiranofaciens*	Kefiran	D‐Glucose, D‐galactose	Variable linkages among glucose and galactose residues	Antimicrobial, hypocholesterolemic, anti‐atherogenic, anti‐colitis	Emulsifier, food additive, food preservative, gelling agent	Tan et al. (2020)
*Pseudomonas* spp., Azotobacter spp.	Alginate	L‐Guluronic acid, D‐mannuronic acid	β‐(1 → 4)	Antimicrobial, antioxidant, inhibit lipid peroxidation of phosphatidylcholine	Thickener, emulsifier, stabilizer, water binding capacity, film formation	Abka‐Khajouei et al. (2022)
*Komagataeibacter* spp., *Acetobacter* spp.	Acetan	D‐Glucose, D‐mannose, L‐rhamnose, D‐glucuronic acid	β‐(1 → 4)‐linked D‐glucose residues with pentasaccharide side chain	Antitumor, antiinflammatory, antioxidative	Food additives, packaging material, encapsulation	Trček et al. (2021)
*Agrobacterium* spp., *Rhizobium* spp., *Pseudomonas* spp.	Succinoglycan	D‐Glucose, D‐galactose, pyruvate, succinate, acetate	β‐Linked galactose and glucose residues with substituents such as pyruvate, succinate, acetate	Antitumor, therapeutic antibacterial agent, reduced splenocyte apoptosis	Thickener, emulsifier, stabilizer in food	Jeong et al. (2022)

*Note:* This table summarizes representative microbial extracellular polysaccharides and their qualitative structural features, reported effects, and food‐related applications. Quantitative properties are not uniformly presented because they vary by strain, cultivation, purification, and application conditions.

Dextran is an α‐glucan primarily composed of glucose with a backbone dominated by α‐1,6 glycosidic linkages (De Bellis et al. 2022). Several LAB species within *Leuconostoc* and *Liquorilactobacillus* can secrete dextran via sucrose‐dependent glucansucrase activity (Llamas‐Arriba et al. 2019). Dextran has been associated with antioxidant, cholesterol‐lowering, and immunomodulatory effects and is widely used to improve rheological properties and texture in fermented foods and baked products (De Bellis et al. 2022).

Mutan is predominantly composed of α‐(1 → 3)‐linked glucose monomers and is primarily synthesized by the bacterium *Streptococcus mutans* . Its water insolubility is attributed to the presence of the α‐1,3 glycosidic bond (Kwon et al. 2016). Mutan is classically produced by *S. mutans* and has also been reported from species within *Leuconostoc* and *Lactobacillus* (Angelin and Kavitha 2020; Yildiz and Karatas 2018). Functionally, mutan contributes to dental biofilm formation by strengthening bacterial adhesion and biofilm stability; because its production is closely linked to sucrose exposure, it is a relevant target in oral health strategies associated with dietary patterns (Bowen and Koo 2011).

Reuteran is a water‐soluble glucan characterized by the presence of α‐1,4 and α‐1,6 glycosidic linkages. Several strains of *Limosilactobacillus reuteri*, which belong to the LAB group, have been documented to synthesize reuteran (Ermann Lundberg et al. 2024). Reuteran has been reported to exhibit prebiotic and shelf‐life‐extending properties. Moreover, in the food industry, reuteran is primarily utilized in baking to improve the softness of baked goods (Korcz and Varga 2021).

Cellulose serves as a primary component of plant cell walls and is also produced by various microorganisms, including algae, bacteria, and fungi (Tang et al. 2024). Cellulose is characterized by a structural composition of β‐1,4‐linked D‐glucose units and is known to be synthesized by *Komagataeibacter* sp. (Singhania et al. 2021). Microbial cellulose and its derivatives are used as thickening, suspending, and stabilizing agents and have also been investigated for food‐packaging applications (Dang et al. 2024; Shen et al. 2023). Additionally, cellulose serves as a suspending or stabilizing agent in food products, and as a fiber‐rich, low‐calorie, and low‐cholesterol dietary supplement. Furthermore, it is utilized as an ingredient in food packaging materials (Netrusov et al. 2023; Wang et al. 2025).

Curdlan is a neutral EPS produced by bacteria belonging to the genera *Alcaligenes*, *Agrobacterium*, and *Paenibacillus* (Chaudhari et al. 2021). It is distinguished by its solubility in alkaline conditions and its insolubility in water (Aquinas et al. 2024). The structural composition of curdlan primarily comprises glucose units that are interconnected through linear β‐(1,3)‐glycosidic bonds (Yuan et al. 2021). Preclinical studies suggest that curdlan may modulate the gut microbiome and show anti‐obesity potential. It is also utilized as a food additive in the dairy industry (Chaudhari et al. 2021).

Levan is a fructan produced by diverse microorganisms, including strains from *Bacillus*, *Halomonas*, *Pseudomonas*, *Aerobacter*, *Streptococcus*, and *Microbacterium* (Kaur and Dey 2023). Levansucrase catalyzes the transfer of D‐fructosyl residues from sucrose, generating levans with a β‐2,6‐linked backbone and β‐2,1 branching (Raga‐Carbajal et al. 2021). Levans have been shown to exhibit hair care benefits, cholesterol‐lowering effects, and prebiotic activity, as well as anti‐irritant, antioxidant, and anti‐inflammatory properties (Domżał‐Kędzia et al. 2019; Öner et al. 2016). Beyond food applications, levans have also been explored in aquaculture, medicine, corrosion inhibition, and sustainable bioprocessing (Öner et al. 2016).

Polygalactan is a HoPS primarily composed of galactose and characterized by repeating galactosyl units (Torino et al. 2015). It has been reported to be secreted by LAB, including *Lactococcus lactis* subsp. *lactis* and *Lactobacillus delbrueckii* subsp. *bulgaricus* (Jurášková et al. 2022).

Collectively, the studies discussed above indicate that the functional diversity of HoPS is governed primarily by glycosidic linkage patterns, backbone architecture, and branching frequency rather than by monosaccharide composition alone. Although many HoPS are composed of glucose or fructose, relatively small structural variations result in distinct physicochemical and techno‐functional properties (Ruas‐Madiedo and De Los Reyes‐Gavilán 2005). Specifically, α‐linked glucans such as dextran and reuteran exhibit excellent water solubility and are widely utilized to improve texture and shelf life in food products, whereas the predominance of α‐1,3 linkages in mutan contributes to water insolubility and biofilm formation (De Bellis et al. 2022; Kwon et al. 2016; Ermann Lundberg et al. 2024). In contrast, β‐linked polymers such as cellulose and curdlan provide structural rigidity, enabling applications in film formation, stabilization, and gelation (Singhania et al. 2021; Yuan et al. 2021). These representative findings demonstrate that linkage type and molecular architecture are major determinants of HoPS functionality in food systems. Nevertheless, direct comparisons among different HoPS remain challenging because their physicochemical properties and food functionalities have often been evaluated using different experimental conditions, analytical methods, and food matrices. Therefore, careful consideration of linkage type and molecular architecture is essential when selecting HoPS for specific food applications, rather than assuming similar functionality based solely on monosaccharide composition.

#### 3.2.2 HePS

Microbial HePS are complex polymers composed of repeating units of three to eight monosaccharides, which may exhibit either branched or unbranched structures (Baruah et al. 2016). HePS are characterized by repeating units of D‐glucose and D‐galactose, along with less common sugars such as L‐rhamnose, arabinose, mannose, xylose, L‐fucose, *N*‐acetyl‐D‐glucosamine, and *N*‐acetyl‐D‐galactosamine (Angelin and Kavitha 2020). In certain cases, non‐carbohydrate components including acetate, glycerate, propionate, pyruvate, succinate, amino acids, L‐glutamate, phosphate, and sulfate may also be incorporated (Kaur and Dey 2023). The synthesis and secretion of HePS are influenced by microbial growth conditions, including medium composition, temperature, pH, oxygen availability, agitation, and growth phase. These conditions can significantly impact the biosynthesis, secretion, quantity, and type (whether ropy or mucoid) of HePS (De Vuyst and Degeest 1999), which in turn influences the monomeric composition, leading to variations in glycosidic linkages (Bajpai et al. 2016).

Xanthan gum, a natural polysaccharide derived from the species *Xanthomonas campestris* , *Xanthomonas phaseoli* , and *Xanthomonas malvacearum*, has various functions, serving as a thickening, stabilizing, and suspending agent in food packaging (Nsengiyumva and Alexandridis 2022; Revin et al. 2023). It is structurally characterized by 1,4‐linked β‐D‐glucose residues, and its anionic properties are attributed to the presence of pyruvic and glucuronic acid groups within its side chains (Kaur and Dey 2023). Xanthan gum has been studied for antimicrobial and antioxidant applications, while its primary food relevance lies in its thickening, stabilizing, and suspending properties (Kumari et al. 2023). Consequently, it has extensive applications across multiple sectors, including agriculture, cosmetics, food production, oil recovery, pharmaceuticals, and textiles (Revin et al. 2023).

Hyaluronic acid is a linear polysaccharide composed of alternating D‐glucuronic acid and *N*‐acetyl‐D‐glucosamine residues linked by β‐(1 → 3) and β‐(1 → 4) glycosidic bonds (Cheng et al. 2023). It is a significant component of the extracellular matrix in both skin and arthritic tissues, and is extensively used across the cosmetics, food, and medical sectors (Liu et al. 2011). Notably, hyaluronic acid is recognized for its capacity to stimulate skin cell activity and support moisture retention and tissue hydration, with applications related to skin and ocular health (Marinho et al. 2021; Papakonstantinou et al. 2012). Hyaluronic acid is produced by various bacterial strains, with *Streptococcus* species and *Pasteurella multocida* being the most prevalent. It has been explored as a texture‐modifying and moisture‐retaining agent and has also shown potential for functional supplementation, in addition to emerging applications in edible coating and biobased films (Cheng et al. 2023).

Gellan gum is an extracellular polysaccharide synthesized by the species *Sphingomonas elodea* and *Sphingomonas pseudosanguinis* through fermentation (Abdl Aali and Al‐Sahlany 2024). Gellan gum consists of tetrasaccharide repeating units containing β‐1,3‐D‐glucose, β‐1,4‐D‐glucuronic acid, and α‐1,4‐L‐rhamnose (Lin et al. 2024). Gellan is characterized by its remarkable gelling properties at low concentrations, exhibiting thermal stability and low pH sensitivity compared to other hydrocolloids (Yamamoto and Cunha 2007). Gellan gum functions as a food additive because of its stabilizing, structuring, and thickening properties, thereby serving as a versatile gelling agent (Kaur and Dey 2023). It is also recognized for its antioxidant and antitumor activities and is used in a diverse array of food products (Abdl Aali and Al‐Sahlany 2024).

Kefiran is primarily secreted by LAB, specifically *Lactobacillus kefiranofaciens* (Tan et al. 2020). Structurally, kefiran is a water‐soluble glucogalactan composed mainly of glucose and galactose units linked through variable glycosidic bonds (Piermaría et al. 2016). It possesses antimicrobial, hypocholesterolemic, antiatherosclerotic, and anti‐colitis properties and has applications as an emulsifier, food additive, food preservative, and gelling agent (Tan et al. 2020).

Alginate is a linear copolymer composed of repeating blocks of β‐D‐mannuronic acid and α‐L‐guluronic acid residues linked through 1,4‐glycosidic bonds (Remminghorst and Rehm 2006). The structural composition of alginate consists of two uronic acid residues, β‐D‐mannuronic acid and its C5 epimer, α‐L‐guluronic acid. These residues are interconnected through 1,4‐glycosidic bonds (Abka‐Khajouei et al. 2022). A diverse array of bacteria from the genera *Azotobacter* and *Pseudomonas* is known to synthesize alginate (Remminghorst and Rehm 2006). Alginate serves multiple functions, including acting as a thickener, emulsifier, stabilizer, water binder, and film former (Zhang et al. 2022). Previous research indicates that alginate can inhibit lipid peroxidation in the context of the anti‐inflammatory and antimicrobial compound phosphatidylcholine (Abka‐Khajouei et al. 2022).

Acetan is secreted by *Komagataeibacter* and *Acetobacter* species and typically contains glucose, mannose, rhamnose, and glucuronic acid; its main chain consists of β‐1,4‐linked D‐glucose residues (Trček et al. 2021). Acetan has been reported to exhibit antitumor, anti‐inflammatory, and antioxidant activities and is used as a food additive and in packaging and encapsulation applications (Trček et al. 2021).

Succinoglycans are produced by Agrobacterium, Rhizobium, and Pseudomonas species (Halder et al. 2017). These polymers contain β‐linked D‐galactose and D‐glucose residues and may include substituents such as pyruvate, succinate, and acetate (Delani et al. 2023). Succinoglycans have been reported to show antitumor and antimicrobial activities and to reduce spleen cell apoptosis in experimental settings, and they are also used as thickeners, emulsifiers, and stabilizers in food applications (Jeong et al. 2022).

Beyond bacterial HePS, EPSs derived from food‐associated cyanobacteria such as *Spirulina* and *Nostoc* have also been reported (de Morais et al. 2015). These cyanobacterial EPSs are typically HePS composed of diverse monosaccharides, often containing uronic acids or sulfated groups, and have been associated with antioxidant and immunomodulatory properties (Liao et al. 2015; Wu et al. 2020). Although cyanobacterial EPSs are not yet produced at the same industrial scale as bacterial HePS, their natural origin and functional potential continue to attract interest for functional food and nutraceutical applications.

Collectively, the studies discussed above indicate that the greater structural complexity of HePS, arising from their diverse monosaccharide composition and the presence of non‐carbohydrate substituents, contributes to a broader range of physicochemical and techno‐functional properties than those of HoPS (Baruah et al. 2016; Kaur and Dey 2023). Variations in repeating‐unit composition, branching architecture, and charged functional groups influence key functional characteristics, including viscosity, gel formation, water‐binding capacity, emulsion stability, and texture modification, thereby determining the suitability of individual HePS for specific food applications (Bajpai et al. 2016; Kaur and Dey 2023). These observations highlight that the functionality of HePS is governed by the combined effects of multiple structural features rather than by any single compositional characteristic. Nevertheless, direct comparisons among reported HePS remain challenging because differences in producing microorganisms, cultivation conditions, purification procedures, and analytical methods can substantially influence polymer composition and functional performance. Therefore, comprehensive structural characterization together with standardized functional evaluation will be essential for establishing robust structure–function relationships and facilitating the rational selection of HePS for targeted food applications.

## 4 Extraction, Recovery, and Production of Microbial EPSs

Before extraction, EPS‐producing LAB should be cultivated under optimized conditions because the growth environment strongly influences EPS yield and polymer‐fraction distribution (Cirrincione et al. 2018). Key fermentation parameters, including the carbon‐to‐nitrogen ratio, pH, and temperature of the culture medium, have been reported to markedly affect EPS biosynthesis, molecular weight, and branching patterns (Jyoti et al. 2024). In general, balanced nutrient levels and slightly acidic to neutral conditions, often around pH 6.0, can increase EPS production, but optimal conditions vary depending on the strain, and under non‐optimized conditions, EPS production of less than 1 g/L has been reported for most LAB (Prete et al. 2021).

After production, EPSs must be isolated and purified to remove process‐related impurities, including medium components, microbial biomass, nucleic acids, and proteins. These steps are essential for both analytical characterization and food‐related applications (Sørensen et al. 2022). To recover EPSs from the cultivation broth, cells are first removed by centrifugation or filtration, and the polymer is then recovered from the cell‐free supernatant (Häffele et al. 2022). Crude EPS preparations can be further deproteinized using proteolytic enzymes or chemical approaches such as trichloroacetic acid to improve purity (Dave et al. 2020).

**FIGURE 2:**
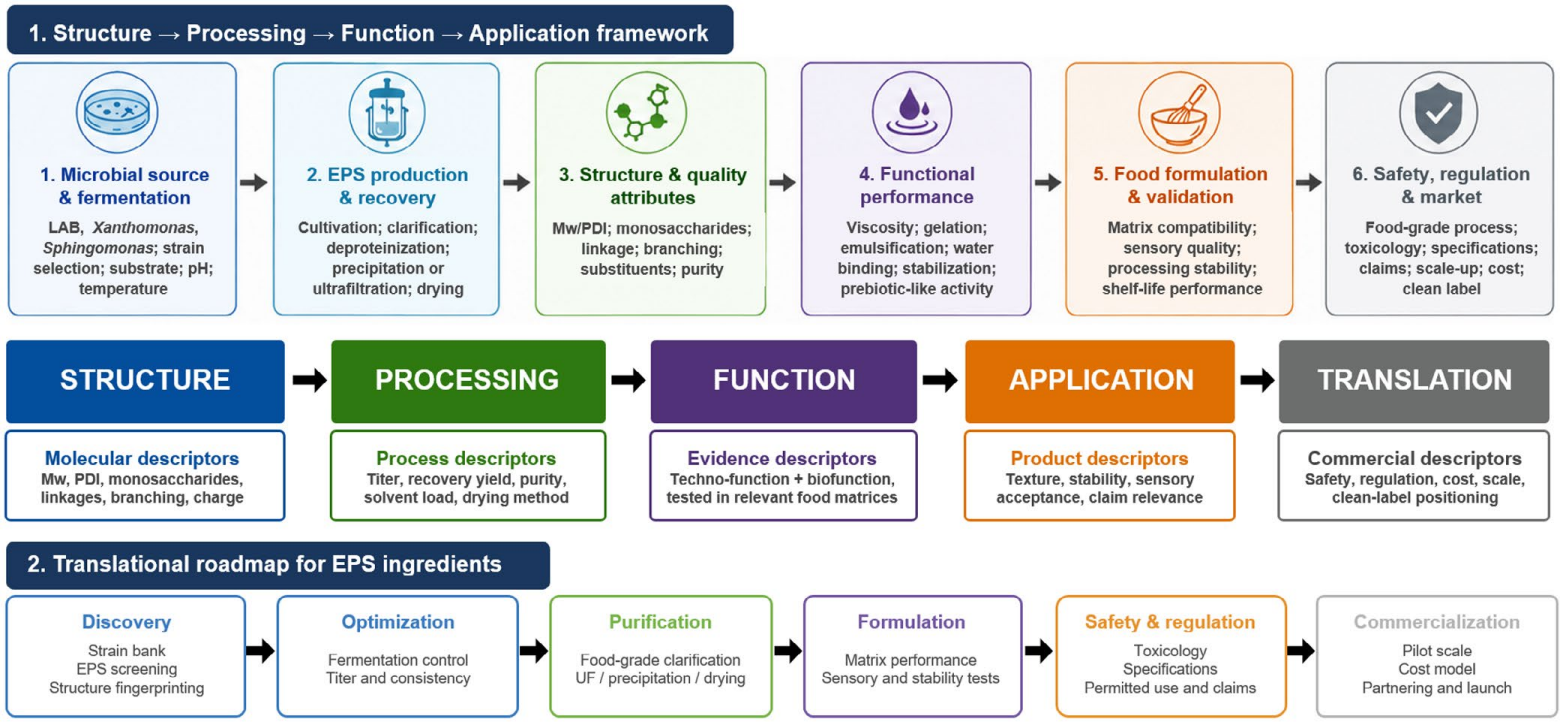
Structure–processing–function–application framework and translational roadmap for microbial exopolysaccharides (EPSs) in food systems. The framework links microbial strain selection and fermentation to EPS structural attributes, downstream processing, techno‐functional and biofunctional performance, food formulation, safety and regulatory evaluation, and commercialization. Key translational checkpoints include titer and consistency, food‐grade recovery, molecular characterization, formulation performance, safety substantiation, regulatory compliance, scale‐up feasibility, and market positioning.

EPS recovery is commonly achieved by solvent precipitation (ethanol or acetone) and/or ultrafiltration (Ziadi et al. 2018). Ultrafiltration using membranes with specific molecular‐weight cutoff (generally 10–500 kDa) provides high recovery and purity but has high initial costs, whereas ethanol precipitation is simple, efficient, and scalable, but it can coprecipitate impurities and requires large volumes of ethanol (Yadav et al. 2024; Ziadi et al. 2018). The resulting precipitate is further purified by dialysis or chromatography and then freeze‐dried or spray‐dried to obtain EPSs (Dave et al. 2020). A general workflow for EPS production and recovery is summarized in Figure 2.

Overall, downstream recovery and purification strategies should be selected according to the intended application by balancing product purity, recovery yield, processing cost, scalability, and food‐grade compatibility (Yadav et al. 2024; Ziadi et al. 2018). For laboratory‐scale characterization, chromatography and dialysis provide high‐purity EPS preparations suitable for structural analyzes, whereas industrial production generally favors cost‐effective approaches such as membrane filtration and solvent precipitation because of their operational simplicity and scalability (Dave et al. 2020). Consequently, integrated process design combining optimized fermentation with application‐oriented downstream processing will be essential to ensure consistent product quality and facilitate the broader utilization of microbial EPSs in food systems.

## 5 Future Prospects

At present, the industrial use of LAB‐derived EPS in the food industry remains substantially lower than that of established plant‐ and animal‐derived polysaccharides. This disparity is primarily attributed to the low yield of LAB‐derived EPS, which restricts its large‐scale industrial application (Zhang, Zhou, et al. 2023). Indeed, LAB‐derived EPS production is typically reported to be below 1 g/L, whereas optimized production of established microbial polysaccharides, such as xanthan gum, gellan gum, dextran, and bacterial cellulose, can exceed 10 g/L (Dev et al. 2022; García‐Ochoa et al. 2000; Prete et al. 2021; Revin et al. 2023; Yang et al. 2015). This benchmark highlights the need to improve LAB‐derived EPS volumetric productivity while maintaining product consistency and functionality, which remain key R&D priorities.

Despite this limitation, LAB‐derived EPS have multiple roles in food applications, including as stabilizers, emulsifiers, and thickening agents, enhancing the sensory attributes, including mouthfeel and flavor (Bibi et al. 2021). They improve oil‐binding capacity, water retention, and viscosity in various food products, including yogurt (Han et al. 2016). These properties are closely associated with EPS structural characteristics, such as molecular weight, glycosidic linkage patterns, and degree of branching, in which higher molecular weight and flexible backbones contribute to viscosity, while branching architecture influences emulsion stability and water‐holding capacity (Badel et al. 2011; Sutherland 2001a). Consequently, application‐driven EPS design linking strain selection and fermentation control to targeted structure–property relationships will be essential for positioning LAB‐derived EPS as reliable hydrocolloids rather than variable fermentation byproducts.

Investigating the biological activities of LAB‐derived EPS, particularly their prebiotic, antioxidant, and anti‐inflammatory properties, is crucial for elucidating the mechanisms underlying their health benefits. Previous research has demonstrated the role of LAB‐derived EPS in preventing gastrointestinal disorders by selectively enhancing the proliferation of beneficial gut microbiota, thus fostering overall gut health, reducing cardiovascular diseases, and enhancing immune system support (Korcz et al. 2018; Rajoka et al. 2020). These health‐promoting properties may provide added value beyond traditional hydrocolloid applications, demonstrating the potential to position EPS as a multifunctional food ingredient rather than simply a texture enhancer (Prete et al. 2021). Although accumulating evidence supports the biofunctional potential of LAB‐derived EPS, further well‐designed human intervention studies are required to substantiate health claims and facilitate their translation into functional food applications (Prete et al. 2021).

From an industrial perspective, the optimization of downstream recovery strategies is a critical factor, as the high energy and solvent requirements for EPS isolation often dictate the overall economic feasibility and commercial viability of the process (Castillo et al. 2015; Freitas et al. 2011). Ethanol precipitation is widely applied due to its simplicity and effectiveness for polymer concentration, whereas membrane‐based ultrafiltration enables improved control over EPS purity and molecular‐weight distribution (Yadav et al. 2024). Therefore, future recovery strategies should be tailored to the intended food application while balancing product quality, economic feasibility, and process scalability, ensuring that the selected process is aligned with both the molecular architecture of the EPS and its desired functional properties (Ziadi et al. 2018). In practice, integrated process design (fermentation → clarification → fractionation → purification → drying) is likely to be necessary to achieve both product performance and cost competitiveness. Future advances will further depend on the development of food‐grade, scalable, and environmentally sustainable manufacturing processes that ensure consistent EPS quality while minimizing production costs (Castillo et al. 2015; Freitas et al. 2011).

The integration of LAB‐derived EPS into common food products has the potential to enhance their organoleptic characteristics, while potentially supporting the development of health‐oriented food (Kaur and Dey 2023). Moreover, the microbial origin and biodegradability of LAB‐derived EPS present a sustainable and health‐oriented alternative to synthetic additives, particularly in light of the increasing consumer preference and interest in clean‐label ingredients (Patel et al. 2010). Several microbial polysaccharides already have established regulatory status as food additives such as xanthan gum (E415) and gellan gum (E418) in the EU, providing useful benchmarks for emerging EPS ingredients, including LAB‐derived EPS (EFSA ANS Panel et al. 2017, 2018). Accordingly, the broader adoption of LAB‐derived EPS as functional food ingredients will require not only continued technological advances but also compliance with food regulatory frameworks and robust substantiation of their safety and functional claims.

Overall, broader adoption of LAB‐derived EPS in the food industry will require coordinated advances across strain engineering, fermentation control, downstream recovery, and regulatory compliance. Further research and development should focus on achieving higher titers, tailored EPS structures, consistent product quality, scalable and low‐impact purification, and robust substantiation of safety and functional claims. With these developments, LAB‐derived EPS could transition from niche ingredients to platform biopolymers supporting the next generation of sustainable, functional, and health‐oriented food products.

## Author Contributions

**Hye‐Bin Lee:** writing – original draft, visualization. **Yu‐Rim Chae:** data curation, writing – original draft. **Yu Ra Lee:** writing – review and editing. **Yoonsook Kim:** writing – review and editing. **Ho‐Young Park:** conceptualization, writing – original draft, writing – review and editing.

## Funding

This work was supported by Korea Food Research Institute, E0210602.

## Conflicts of Interest

The authors declare no conflicts of interest.

## Data Availability

Data sharing not applicable to this article as no datasets were generated or analysed during the current study.
